# Molecular and Cellular Mechanisms of Itch in Psoriasis

**DOI:** 10.3390/ijms21218406

**Published:** 2020-11-09

**Authors:** Eriko Komiya, Mitsutoshi Tominaga, Yayoi Kamata, Yasushi Suga, Kenji Takamori

**Affiliations:** 1 Juntendo Itch Research Center (JIRC), Institute for Environmental and Gender Specific Medicine, Juntendo University Graduate School of Medicine, 2-1-1 Tomioka, Urayasu, Chiba 279-0021, Japan; ekomiya@juntendo.ac.jp (E.K.); ykamata@juntendo.ac.jp (Y.K.); ktakamor@juntendo.ac.jp (K.T.); 2 Anti-Aging Skin Research Laboratory, Juntendo University Graduate School of Medicine, 2-1-1 Tomioka, Urayasu, Chiba 279-0021, Japan; ysuga@juntendo.ac.jp; 3 Department of Dermatology, Juntendo University Urayasu Hospital, 2-1-1 Tomioka, Urayasu, Chiba 279-0021, Japan

**Keywords:** cytokines, HPA axis, itch, neurogenic inflammation, neuropeptides, pruritus, psoriasis, vascular system

## Abstract

Itch (or pruritus) was not previously recognized as a serious symptom of psoriasis. However, approximately 60–90% of psoriatic patients with pruritus have stated that it deteriorates their quality of life. Since conventional antipruritic therapies, such as antihistamines, only exert limited effects, the establishment of a treatment option for itch in psoriasis is urgently needed. Although a definitive drug is not currently available, various itch mediators are known to be involved in pruritus in psoriasis. In this review, we describe the clinical features of pruritus in psoriasis, classify a wide range of itch mediators into categories, such as the nervous, immune, endocrine, and vascular systems, and discuss the mechanisms by which these mediators induce or aggravate itch in the pathophysiology of psoriasis.

## 1. Introduction

Psoriasis is one of the most common inflammatory skin diseases and affects approximately 1–3% of the general population worldwide [1]. Although the term “psoriasis” comes from the Greek word “psora”, which means itch [2], psoriatic itch has often been underappreciated and overlooked as a symptom and is not considered in disease management [3,4,5]. However, recent studies demonstrated that approximately 60–90% of patients with psoriasis have pruritus [1,6,7,8,9,10], and this value may change depending on the country, region, or race. In addition, the severity of itch in psoriatic patients has been thought to be no higher than that of atopic dermatitis; however, a recent meta-analysis of data from 22 clinical trials revealed no significant differences in the baseline severity of itch between these diseases [11].

Accumulating evidence has shown that psoriatic patients consider pruritus as not only the most common subjective sensation [12,13], but also as one of the most bothersome symptoms of this disease [9,13,14,15]. However, despite its importance, effective therapy for itch in psoriasis has not yet been established. This may be due to the lack of awareness of itch and the large number of itch mediators involved in this disease. Therefore, this review focuses on itch in psoriasis, describes its clinical characteristics, and discusses the molecular and cellular mechanisms of itch in psoriasis based on its pathophysiology.

## 2. Clinical Characteristic of Itch in Psoriasis

The Psoriasis Area and Severity Index (PASI) and the Physician’s Global Assessment (PGA) are the most commonly used assessment tools to clinically evaluate the severity of psoriasis [4]. However, these methods are not suitable for assessments of itch. One of the most common assessment tools for the evaluation of itch is a visual analog scale (VAS). VAS is an evaluation method that uses a drawn 100 mm horizontal line as a graphic tool, and patients are asked to show points that correspond to the severity of itch (from a minimum value of 0 to a maximum value of 10) [16]. Although itch in psoriatic patients varies in severity from mild to severe [10], the majority of patients with psoriasis score between 4.2 and 6.4 points on VAS. These values indicate that patients with psoriasis generally have itch of moderate severity [6,10,17].

Although psoriasis is more prevalent in men, it was previously shown to be more frequent and severe in women than in men [6,12]. Itch may affect any body area, but more commonly develops on the lower limbs and body trunk, followed by the scalp [1,6,10,18,19]. In contrast, it is rarely present on the face and neck [1,10]. Furthermore, in approximately 75% of patients with psoriasis, itch appears on a daily basis and with a prolonged duration [1,6,10]. Moreover, as the severity of psoriasis increases, not only itch, but also pain or sometimes a burning sensation may occur [20,21]. The duration of psoriasis, the marital status of patients, and a family history of psoriasis or atopic dermatitis were found to have no effect on the severity of itch [1,6,10,12,18].

Several factors, including winter, night time, hot water, exercise, or a negative mood, have been shown to aggravate itch in psoriasis, whereas sleep and cold showers alleviated it [10]. These findings indicate that skin dryness, an elevated temperature, sweating, and psychological stress are exacerbating factors of pruritus in psoriasis [4]. Since the majority of these factors are considered to aggravate not only itch, but also the pathology of psoriasis, they have been suggested to contribute to the relationship between the severity of itch and symptoms of psoriasis [7,13,18,19]. Similarly, severe itch commonly emerges when skin lesions appear or psoriatic plaques expand, and significant relief from pruritus is generally associated with the complete resolution of psoriatic lesions [1,10]. These findings also support the positive correlation between psoriatic symptoms and the severity of itch. However, this correlation has not been observed in all studies [10,22]. Itch in both the lesional and non-lesional skin of patients with psoriasis questions the veracity of this correlation.

In addition to the mechanism by which pruritus becomes worse with the aggravation of psoriasis, scratching exacerbates the pathological conditions of psoriasis. A previous study reported that trauma caused by scratching behavior resulted in new psoriatic lesions in non-lesional areas [4], which is referred to as the Koebner phenomenon [23,24] and is considered to play a pivotal role in the itch-scratch cycle in the pathology of psoriasis [4].

Pruritus and the pathology of psoriasis are interrelated in a complex manner. Therefore, a more detailed understanding of itch in psoriasis may contribute to the development of more effective treatments for the pathological features of psoriasis.

## 3. Itch Transmission Pathway

Itch affects the skin and some mucosa, but not internal organs, such as the stomach. Therefore, this sensation is induced by the excitation of peripheral sensory nerve fibers (free nerve endings), which are distributed in the skin, by chemical stimuli (caused by chemical mediators) or mechanical stimuli (caused by insects or parasites) [25].

Electrophysiological analyses revealed that peripheral sensory nerve fibers which convey itch are broadly divided into unmyelinated C-fibers and thinly myelinated Aδ-fibers [26,27]. Unmyelinated C-fibers mainly transmit the itch signal and more slowly than myelinated Aδ-fibers. Unmyelinated C-fibers are further subdivided into peptidergic and non-peptidergic fibers [27,28,29]. The former comprise neuropeptides, such as substance P (SP) and/or calcitonin gene-related peptide (CGRP), whereas the latter express the purinergic receptor P2X3 and isolectin B4 [29]. Aδ-fibers respond to histamine- as well as cowhage spicule-induced itch, and more strongly contribute to cowhage-induced itch than to that by histamine or capsaicin (a well-known algesic substance) [26].

This excitatory information from the sensory nerve fibers described above conveys itch signals by various neurotransmitters to secondary neurons distributed in the dorsal horn of the spinal cord. Information from the spinal cord then passes through the thalamus and posterior head cortex, and is ultimately processed at multiple areas in the brain [27,30,31].

## 4. Pathophysiology of Itch in Psoriasis

**Figure 1:**
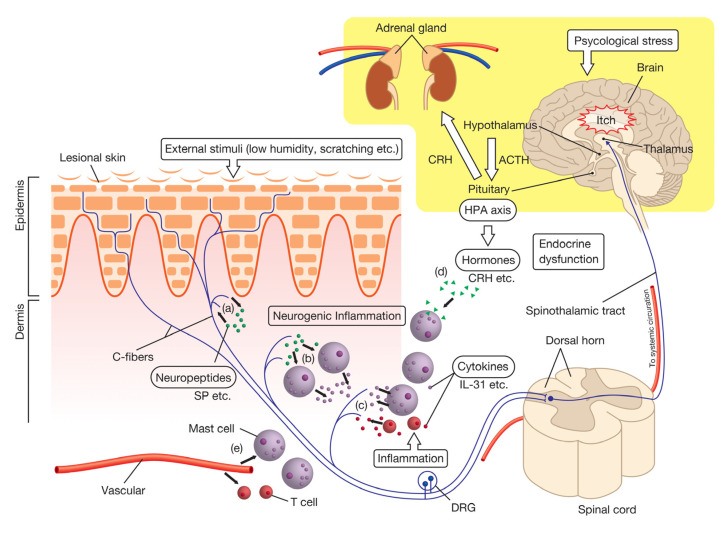
Mechanisms from the triggering to sensing of itch in psoriasis. Itch in psoriasis is triggered or exacerbated by external stimuli, lesional inflammation, and psychological stress. External stimuli, such as scratching behavior, promote the secretion of neuropeptides from nerve endings (axon reflex). Neuropeptides act on C-fiber neurons both (**a**) directly (by acting retrogradely on neurons) and (**b**) indirectly (via neurogenic inflammation) to convey itch signals to the central nervous system (spinal cord and brain). In lesional skin, (**c**) various immune cells, such as mast cells or T cells, secrete a number of cytokines, which mainly aggravate itch by enhancing inflammatory responses, such as mast cell degranulation. Some of these responses also elicit itch by directly acting on nerve endings. In addition, the HPA axis, which is stimulated by psychological stress promotes hormone secretion (such as CRH) to lesional skin. These hormones (**d**) are also involved in the aggravation of itch, mainly by mast cell degranulation. The vascular system also aggravates itch, mainly by (**e**) recruiting immune cells to lesional sites via angiogenesis or increasing vascular permeability. (Abbreviations: CRH; Corticotropin-Releasing hormone, DRG, Dorsal root ganglion, HPA; Hypothalamic–pituitary–adrenal, IL-31; Interleukin-31, SP; Substance P).

Since psoriasis causes skin lesions, the majority of studies on itch have focused on the pathology of peripheral skin lesions in psoriatic patients. A number of itch mediators have been identified based on the pathological features of psoriasis (Table 1). However, the factors responsible remain unknown. The effects of antihistamines (histamine-H_1_ receptor antagonists) on itch in psoriasis are generally considered to be limited [10,32,33,34]. Therefore, various factors are intricately intertwined and coordinately associated in the induction of itch in psoriasis. We herein describe itch-related molecules in psoriasis by classifying them into six categories, such as the nervous, immune, endocrine, and vascular systems (Figure 1).

### 4.1. The Nervous System

Since all itch stimuli in lesional skin are ultimately transmitted though nerves to the brain, the nervous system plays a crucial role in the mechanisms underlying itch. Various types of neuropeptides, which are secreted from nerve endings, directly or indirectly act on nerves to induce or increase the severity of itch in psoriasis. In addition, opioid receptors, which are expressed on sensory nerves, their ligands, and the transient receptor potential (TRP) family coupled with many types of pruriceptors may modulate the degree of itch in psoriasis.

#### 4.1.1. Neuropeptides

Neuropeptides is a general term for endogenous active peptides that are expressed in neurons and involved in neurotransmission [78]. In the brain, they play crucial roles in a number of physiological functions, such as learning and memory or sleep. They are generally released by an axon reflex from nerve endings of peptidergic nerve fibers into peripheral tissue. The neuropeptides released cause an inflammatory response, such as vasodilation or mast cell degranulation, which is called neurogenic inflammation (a series of inflammatory responses triggered by the activation of primary sensory neurons and the subsequent release of inflammatory neuropeptides) [79]. Degranulated mast cells release histamine, proteases, and pro-inflammatory mediators, which induce itch. In addition, some neuropeptides retrogradely act on nerves and transmit itch sensation directly to the spinal cord. Therefore, some neuropeptides aggravate pruritus by both direct and indirect effects due to neurogenic inflammation in nerve fibers. We herein describe the effects of some neuropeptides that have been reported to correlate with the severity of pruritus in psoriasis.

(1) Substance P (SP)

Substance P (SP) is a sensory undecapeptide of the tachykinin family that is distributed widely in the central and peripheral nervous systems [80]. When applied intradermally to the skin, SP elicits itch in humans [37] and mice [38]. Many studies have demonstrated increases in SP levels with psoriasis, and these findings have been summarized in a review by Saraceno et al. [80]. Furthermore, the number of SP-immunoreactive fibers in perivascular areas was found to be higher in psoriatic patients with than in those without itch [35]. Another study reported that the number of SP-immunoreactive fibers in lesional skin correlated with the severity of pruritus [81]. Chang et al. found that keratinocytes in the psoriatic plaques of patients with pruritus more strongly expressed neurokinin-1 receptor (NK-1R), a receptor of SP [7]. Moreover, NK-1 inhibitors, such as aprepitant and serlopitant, were effective against chronic itch through their peripheral and/or central effects [82,83]. It has been thought that the receptor of SP was only NK-1R; however, more recently, this neuropeptide was shown to also bind to human MrgprX2, mouse MrgprB2, expressed in mast cells, and mouse MrgpA1, expressed in nerve fibers [84]. Although this peptide induces itch through mast cell degranulation and sensory neuron activation [85], behavioral experiments revealed that sensory nerves are the main drivers of SP-induced itch in mice [38]. In 2017, scratching behavior and activation of the cultured dorsal root ganglia of mice by SP were both found to be more dependent on MrgprA1 than on NK-1R [85]. Since changes in the expression levels of MrgprA1, MrgprB2 in mouse, or MrgprX2 in humans with psoriatic lesions currently remain unknown, further investigations are needed. In addition to these receptors, neural endopeptidase (NEP) and dipeptidyl peptidase IV (DPPIV) have been identified as enzymes that are capable of controlling the bioactivity of SP [75,86]. A decrease in the former [81] and increase in the latter [36,75] have been reported with psoriasis, which may contribute to the aggravation of itch [75]. The relationship between SP and DPPIV is discussed later (Section 4.6.1).

(2) Calcitonin Gene-Related Peptide (CGRP)

Calcitonin gene-related peptide (CGRP) is a neuropeptide that consists of 37 amino acids. It is widely expressed in both the central and peripheral nervous systems [80]. In peripheral tissue, it is located in both unmyelinated peptidergic C-fibers and myelinated Aδ-nerve fibers and functions as a thermal pain-modulating neurotransmitter [80,87]. Although it currently remains unclear whether this peptide directly induces itch in humans [88], the genetic ablation of CGRP-expressing sensory neurons was found to ameliorate itch in mice [39]. Based on these findings, CGRP might be involved in enhancement of itch. Higher plasma levels of CGRP have been found in psoriatic patients with pruritus, and these levels correlated with the severity of pruritus in selected subgroups of these patients [9,15]. Moreover, CGRP receptors (CGRPR) have been detected in psoriatic skin and associated with the severity of pruritus, thereby supporting its potential role in the development of itch in psoriasis in one study [7], but not in another, suggesting that careful assessments are still needed for these markers [4].

(3) Neuropeptide Y (NPY)

A previous study reported that plasma levels of neuropeptide Y (NPY) were significantly lower in psoriatic patients with than in those without pruritus [40]. Furthermore, NPY signaling was recently shown to constitutively suppress mechanical itch in normal mice by inhibiting NPY receptor 1-expressing neurons, which are required for mechanical itch transmission, in the dorsal horn of the spinal cord [25]. These findings indicated that reduced levels of NPY expression psoriasis aggravated mechanical itch; however, further investigations are needed to confirm this.

#### 4.1.2. Opioid Ligands and Their Receptors

**Figure 2:**
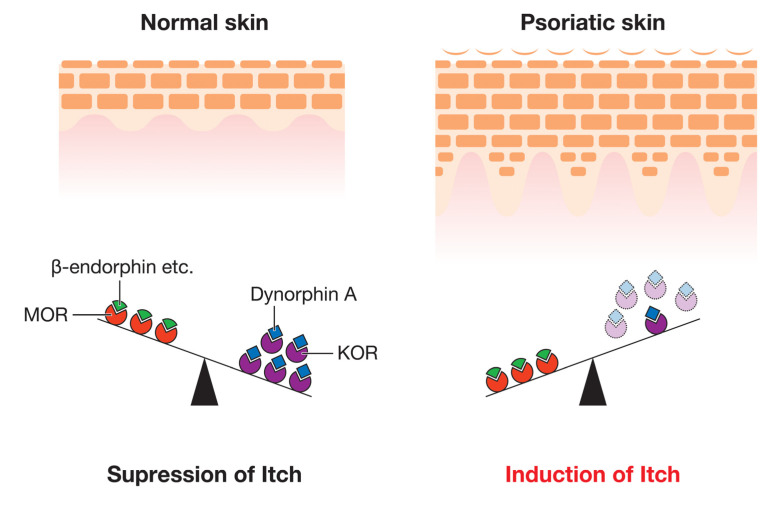
Changes in the expression of opioid receptors and their ligands in psoriatic lesions. In normal skin (**left**), itch is constitutively suppressed when KOR and its ligand dynorphin A, which attenuate itch, are strongly expressed and MOR and its ligand β-endorphin, which are involved in the induction of itch, are also expressed. However, in the lesional skin of patients with psoriasis (**right**), although the expression of MOR and β-endorphin remain unchanged, that of KOR and dynorphin A decreases. This imbalance between KOR and MOR controls itch in psoriasis. (KOR; κ-opioid receptor, MOR; μ-opioid receptor).

Opioid receptors are classified into four types: the μ-opioid receptor (MOR), δ-opioid receptor (DOR), κ-opioid receptor (KOR), and nociceptin opioid (NOP) receptor [89]. These receptors play crucial roles in pain modulation, and agonists of these receptors are used for analgesia [43,90]. However, despite the uniform analgesic effects of these receptors, MOR and KOR have been shown to exert opposite effects on itch; MOR induces itch through its activation by MOR ligands (such as β-endorphin), while the interaction between KOR and its ligand (dynorphin A) suppresses it [43]. We previously demonstrated that the expression levels of KOR were lower in the lesional epidermis of psoriatic patients with pruritus than in healthy controls, whereas those of MOR were similar [41]. These findings indicate that the imbalance between the activation of these opioid pathways is due to the severity of pruritus in psoriasis (Figure 2). Regarding ligands, the expression level of dynorphin A was significantly decreased in psoriatic patients with pruritus, whereas that of β-endorphin was unchanged [41]. In another study, similar findings were obtained for the expression of opioid receptors in the skin, while the expression of KOR in non-lesional skin was significantly higher than in control skin [91]. Another study reported that serum levels of β-endorphin were higher in patients with psoriasis than in healthy controls [42]. Moreover, scratching behavior in imiquimod-induced psoriasis model mice was suppressed by central and peripheral MOR antagonists or a central KOR agonist [89]. Based on these findings, peripheral MOR, central MOR, and central KOR appear to be at least partially involved in the regulation of pruritus in psoriasis.

#### 4.1.3. TRP Cation Channel Subfamily (TRP Channels)

TRP channels are nonselective calcium-permeable cation channels that comprise the TRP ion channel superfamily [92]. Twenty-eight TRP family members have been identified in mice and 27 in humans, and have been classified into six subgroups based on sequence homology (TRP canonical (TRPC), TRP vanilloid (TRPV), TRP melastatin (TRPM), TRP ankyrin (TRPA), TRP polycystin (TRPP), and TRP mucolipin (TRPML)) [93]. TRP ion channels may have functions in itch through their coupling with pruritogenic G-protein-coupled receptors (GPCRs), such as histamine receptors and serotonin receptors [93,94].

Previous studies demonstrated that the gene expression levels of TRPV1, TRPM8, and TRPV3 were significantly elevated in pruritic skin with psoriasis [4,58]. Among these genes, the overexpression of the TRPV1 gene correlated with the severity of pruritus in psoriasis [92], and the interaction between TRPV1-immunoreactive fibers and dendritic cells has been shown to regulate the IL-23/IL-17 pathways and promote psoriatic inflammation [59]. In addition, prolonged exposure to capsaicin, an agonist of the TRPV1 channel, resulted in desensitization in an extracellular calcium-dependent manner and reduced the severity of psoriatic itch [95]. These findings suggest that TRPV1 plays an essential role in the transduction of itch in psoriasis.

Lee et al. recently reported that another TRP ion channel, TRPC4 may play a pivotal role in psoriatic itch [94]. Using imiquimod-induced psoriasis model mice, they demonstrated that TRPC4 was expressed in a subset of peptidergic neurons expressing TRPV1 that innervate the skin. They also examined the effects of the local intradermal administration of the specific TRPC4 inhibitor ML204 on itch and inflammation in the psoriatic mouse model, and found that this inhibitor significantly attenuated itch in these mice [94]. Although further studies are needed, specific TRPC4 inhibitors may be effective in the treatment of itch and inflammation in psoriasis.

#### 4.1.4. Nerve Growth Factor (NGF)

Nerve growth factor (NGF) is a secretary protein that belongs to the neurotrophic factor family, and is involved in the regulation of growth, proliferation, survival, and maintenance of sympathetic and sensory afferent neurons (peptidergic fibers) [29,96]. In healthy subjects, NGF sensitizes nociceptors for cowhage-, but not histamine-induced itch, indicating that this sensation is mediated by polymodal nociceptors [45]. NGF binds to two receptors: tropomyosin-receptor kinase A (TrkA) and low affinity receptor p75 [97]. In psoriasis, immunoreactivity for NGF is strong throughout the entire epidermis, the expression of TrkA is elevated in basal keratinocytes and dermal nerves, and the expression levels of these proteins correlate with the severity of pruritus [35]. Furthermore, NGF expression levels were significantly higher in lesional pruritic skin than in non-lesional skin with psoriasis [44]. The topical TrkA kinase inhibitor CT327 was found to be effective therapy for pruritus in patients with psoriasis [98]. These findings indicate that the NGF-TrkA axis plays an important role in the aggravation of itch in psoriasis.

#### 4.1.5. Sensory Nerve Fiber Density

Hyperinnervation is considered to potentiate the itch sensation. NGF and IL-31, which are strongly expressed in the lesional skin of psoriasis [35,46], have been shown to promote the growth of sensory nerves [96,99], suggesting that the elongation and branching morphogenesis of epidermal nerve fibers are involved in the hypersensitivity of itch in psoriasis. However, some studies reported an increased nerve density in psoriatic skin [35], whereas others found no correlation [41] or even a reduction in nerve density [100]. This disparity may be explained by the duration, location, and progression of lesions in the samples obtained and differences in nerve density measurements. In a recent study on an imiquimod-induced psoriasis mouse model, the total number of nerve fibers crossing the dermal–epidermal junction was not significantly different from that in normal mice, while the proportion of non-peptidergic nerve fibers was higher. Furthermore, the inhibition of neurturin, an important neurotrophic growth factor for non-peptidergic sensory neurons, significantly attenuated the scratching behaviors of imiquimod-induced psoriasis model mice [101]. Moreover, heightened sensitization of existing itch-selective nerve fibers may occur, in parallel with changes in nerve density [102]. Collectively, these findings indicate that further studies are needed to clarify the contribution of intradermal nerve density to itch in psoriasis.

### 4.2. The Immune System

Various immune cells, such as mast cells or T cells, secrete many cytokines that indirectly aggravate itch by increasing inflammatory responses. Several cytokines have also been shown to induce itch. Mast cells secrete various itch mediators through degranulation, thereby contributing to the aggravation of itch. However, the contribution of the immune system, including cytokines, to pruritus in psoriasis has not yet been examined in as much detail as that of other diseases, such as atopic dermatitis. We herein describe the most recent immunological findings obtained on itch in psoriasis.

#### 4.2.1. Cytokines

A recent study demonstrated that a number of cytokines are involved in the pathogenesis of psoriasis. Even though some of them are still not clearly associated itch, we herein discuss the mechanisms of itch in psoriasis from the aspect of cytokines [56].

(1) Itch-Mediating Cytokines

(1.1) Interleukin-31 (IL-31)

Interleukin-31 (IL-31) is one of cytokines that induce itch [48], and has also been shown to play a role in atopic dermatitis [103]. Recent studies demonstrated that IL-31 gene transcription [58], its serum level [47], and the number of IL-31-immunoreactive mast cells at lesional sites [46] were elevated in patients with psoriasis. Furthermore, serum IL-31 levels were significantly reduced after narrowband ultraviolet B (UVB) phototherapy resulting in a substantial reduction in psoriatic patients with pruritus [47]. In contrast, a correlation was not observed between serum IL-31 levels and the severity of pruritus in psoriatic patients [104]. Therefore, although there are disparities in the literature, IL-31 appears to contribute to the induction of itch in psoriasis [105].

(1.2) Thymic Stromal Lymphopoietin (TSLP)

Similar to IL-31, thymic stromal lymphopoietin (TSLP) is an itch-inducible cytokine [51]. It has been shown to play a role in itch of atopic dermatitis since its expression levels in keratinocytes were higher in patients with atopic dermatitis than in healthy subjects [51]. A recent study revealed that this cytokine plays an important role in the pathogenesis of scalp psoriasis [106]. Since elevated expression levels of TSLP in the epidermis [49] and increased TSLP serum levels [50] have been reported in patients with psoriasis, this cytokine appears to be partially involved not only in the pathogenesis of psoriasis, but also in the induction of itch in psoriasis.

(1.3) IL-2

Nakamura et al. reported that the number of IL-2-immunoreactive cells was higher in the pruritic lesions of psoriasis than in non-pruritic lesions [35]. Immunotherapy with this cytokine for metastatic cancer is associated with psoriasis-like dermatological complications, including pruritus, and exacerbated the psoriatic pathology of cancer patients with a history of psoriasis [107]. IL-2 has also been identified as a potent activator of a discrete population of cutaneous C-polymodal nociceptors, which are chemo-sensitive to endogenous inflammatory mediators [52]. A previous study reported that IL-2 exerted a rapid weak pruritogenic effect, which appeared to be followed by an inflammatory response, in healthy human subjects [53]. Furthermore, a single intradermal injection of IL-2 induced local pruritus and erythema with dermal T-cell infiltrates in atopic dermatitis patients and healthy controls [54]. These findings support IL-2 at least partly contributing to the induction of itch in psoriasis [35].

(2) Cytokines Involved in the Pathogenesis of Psoriasis

(2.1) IL-17

In the context of an adaptive immune response, psoriasis has historically been characterized as a T helper type (Th) 1-mediated disease. More recently, Th17-mediated immunity was shown to play a pivotal role in the pathogenesis of psoriasis [21]. IL-17 is known to directly and indirectly act on neurons in the dorsal root ganglion (DRG) or spinal cord and enhance nociceptive effects [55]. Therefore, it currently remains unclear whether IL-17 is directly involved in induction and/or enhancement of itch; however, it may play a role in psoriatic itch by increasing the sensitivity to sensory reception, in addition to its effects on the pathogenesis of psoriasis. Monoclonal antibodies targeting IL-17 have been shown to attenuate the severity of pruritus in psoriasis by >70% [108,109,110].

(2.2) IL-22

IL-22 is a member of the IL-10 family. This cytokine is produced by various cells, such as Th17 and Th22, and plays a role in homeostasis in the mucosa and barrier organs with the pathology of psoriasis [56]. In patients with psoriasis, IL-22 levels were found to be elevated in serum and skin lesions and positively correlated with disease severity [102,111]. The expression of IL-22 was stronger in the scalp than in other anatomical sites [102,112]. Based on the finding showing that GRP expression was increased in dermal immune cells, afferent nerves, and neurons in skin-innervating DRG of IL-22 transgenic atopic dermatitis murine model and IL-22-treated human keratinocytes [57], IL-22 may be involved in the activation of the GRP receptor pathway, an itch-specific pathway [113,114]. These findings imply that IL-22 plays some roles in the enhancement of itch in psoriasis.

(2.3) IL-23

IL-23 is closely associated with the pathogenesis of psoriasis since it maintains the cytokine milieu required for the survival of Th17 cells [56,115]. An imiquimod-induced psoriasis model was found to possess a large population of nociceptive sensory neurons in close proximity to IL-23-producing cells. The ablation of these nociceptors resulted in the failed production of IL-23, and a subsequent decline in inflammatory responses, indicating that IL-23-induced inflammation is associated with noxious skin sensations [59]. IL-23A gene transcription levels were previously shown to be higher in the skin of patients with psoriasis than in healthy controls [58]. Ustekinumab also has potential as an agent to alleviate pruritus in psoriasis [116].

(2.4) IL-26

IL-26 is a member of the IL-10 family [117,118]; however, its functions in humans have not yet been clarified because it is not present in mice or rats [119]. We recently reported that erythema symptoms were more severe in a human IL-26 transgenic imiquimod murine model than in control imiquimod model mice [60]. Our in vivo and in vitro assays revealed that IL-26 promoted angiogenesis by upregulating FGF2 and FGF 7 [60]. Since the FGF-FGFR1 axis is essential for the development of TrkA-positive unmyelinated neurons that transmit pain and itch sensations [61], IL-26 may play a role in the sensory neuronal development by promoting the expression of these FGFs during the pathogenesis of psoriasis. These findings also suggest that anti-IL-26-neutralizing monoclonal antibodies, which alleviate IL-26-derived psoriatic symptoms [120], attenuate pruritus in psoriasis.

#### 4.2.2. Mast Cells and Gamma-Amino Butyric Acid (GABA)-Expressing Inflammatory Cells

Mast cells are mostly located perivascularly in close proximity to nerve fibers. Previous studies reported elevated numbers of mast cells in the papillary dermis of pruritic lesions [35,121]. Although mast cells release histamine, no significant differences were observed in plasma histamine levels between psoriatic patients with or without pruritus [34]. This finding may explain why less than 20% of psoriatic patients claimed that oral antihistamines were effective at attenuating pruritus [18]. In spite of these findings, mast cells appear to be involved in the induction or aggravation of psoriatic itch, since they also release proteases and pro-inflammatory mediators that induce itch in psoriasis [63,102].

An elevated number of gamma-amino butyric acid (GABA)-positive macrophages and GABA (A) receptor-expressing lymphocytes have been observed in psoriatic patients, and the number of inflammatory cells correlated with the severity of pruritus [122]. Thus, GABA may modulate the activity of various immune cells and potentially stimulate them to secrete other pruritogenic mediators, such as IL-31 or IL-2 [1].

#### 4.2.3. Janus Kinase-Signal Transducer and Activator of Transcription (JAK-STAT) Pathway

The majority of cytokines rely on the Janus kinase-signal transducer and activator of transcription (JAK-STAT) pathway because their receptors lack receptor-intrinsic kinase activity and instead transmit their signals through receptor-associated Janus kinases and the activation of stats, which are transcription factors [123,124]. Indeed, all of the cytokines described in this review, except IL-17, have been reported to transmit their signals via JAK-STAT pathway [125,126,127,128,129,130]. Furthermore, the expression and activation of STAT1 and STAT3 were found to be stronger in the lesional skin of psoriasis than in non-lesional skin [131,132]. These findings suggest that the inhibition of JAKs is a powerful and more profound antipruritic treatment than that with a single monoclonal antibody [123]. Consistent with these findings, the JAK inhibitor tofacitinib exerted antipruritic effects independent of improvements in erythema, induration, and scaling which were measured by PGA [133].

### 4.3. The Endocrine System

The hypothalamic–pituitary–adrenal (HPA) axis is one of the major sources of the neuroendocrine system and consists of a complex set of direct influences and feedback interactions of several hormones from three components: the hypothalamus, pituitary gland, and adrenal glands. This axis is stimulated by psychological stress. Since psychological stress is one of the exacerbating factors of symptoms of psoriasis, the involvement of the HPA axis in the inflammatory responses of psoriasis has been investigated in detail [134,135]. However, the role of the HPA axis in pruritus in psoriasis remains unclear. In this section, we discuss the involvement of the HPA axis in psoriatic itch.

#### 4.3.1. Corticotropin-Releasing Hormone (CRH)

Corticotropin-releasing hormone (CRH) is the first hormone of the HPA axis that is released from the hypothalamus in response to stress, and then induces the downstream release of peptides from the pituitary gland, such as adrenocorticotrophic hormone (ACTH). In addition to the hypothalamus, CRH is expressed in the skin, particularly in the epidermis, sweat glands, and hair follicles, and the significant upregulation of its expression has been demonstrated in psoriatic skin lesions [62]. This hormone promotes mast cell degranulation and increases vascular permeability, suggesting that stress evokes mast cell degranulation, which is mediated by the release of CRH [63,64]. In stressed mouse models, the administration of CRH antiserum and an anxiolytic/antipsychotic significantly suppressed the degradation of mast cells [136,137]. CRH antagonists have been attracting increasing attention as potential therapeutics in the context of neurogenic inflammation [138,139]. CRH antagonists may be used as therapeutic agents for pruritus in psoriasis induced by psychological stress.

#### 4.3.2. α-Melanocyte-Stimulating Hormone (α-MSH)

α-melanocyte-stimulating hormone (α-MSH) is a hormone that is produced by the cleavage of ACTH, a second hormone in the HPA axis that is secreted from the pituitary gland. The expression levels of α-MSH and ACTH were previously shown to be higher in the lesional skin of psoriatic patients than in controls, but did not significantly differ from each other [62]. An intradermal injection of α-MSH induced itch-related scratching behavior in mice that was subsequently inhibited by a H_1_ histamine receptor [65] or thromboxane A2 (TXA_2_) antagonist [66]. In an atopic dermatitis murine model, the expression of α-MSH was mainly observed in keratinocytes, and scratching responses in these mice were attenuated by an antagonist of melanocortin 1 receptor (MC1R), which is one of the receptors of α-MSH [66]. In addition, the production of TXA_2_, an α-MSH-induced itch modulator, was decreased by each small interference RNA (siRNA) for MC1R and melanocortin 5 receptor (MC5R) in keratinocytes [66]. These findings indicate that α-MSH-MC1R/MC5R axis is also involved in the induction of psoriatic itch induced by psychological stress.

### 4.4. The Vascular System

Vascular abnormalities are frequently observed in psoriatic lesions, indicating the importance of changes in the dermal vasculature in the pathogenesis of psoriasis [60,74]. However, data on the direct involvement of this system in psoriatic itch are limited, compared with other systems, such as the nervous and immune systems. The vascular system appears to mainly induce itch indirectly by recruiting a large number of immune cells to lesional sites by angiogenesis and increasing vascular permeability. We herein present some candidates as itch mediators from the aspect of the vascular system.

#### 4.4.1. Vascular Endothelial Growth Factor (VEGF)

Vascular endothelial growth factor (VEGF) is a key factor promoting angiogenesis and vasculogenesis. Angiogenesis induced by VEGF has been shown to play a key role in psoriasis [140], and serum VEGF levels are elevated in psoriatic patients and correlate with disease severity [68]. A recent study reported that serum levels of VEGF in mycosis fungoides and Sézary syndrome positively correlated with markers of pruritus (serum immunoglobulin E and NGF) and VAS scores [141]. VEGF expression levels were found to be higher in the lesional skin of psoriatic patients with pruritus than in those without pruritus [67]. This increased expression was also observed in the imiquimod-induced psoriasis mouse model [67]. Moreover, it has been reported that keeping of epidermis-specific VEGF transgenic mice for 25 weeks to induce psoriasis-like pathology, leads to increases in the frequency of scratching behavior [67]. Furthermore, a selective inhibitor of VEGF receptor tyrosine kinases 1–3 (axitinib) suppressed scratching behavior in imiquimod-induced psoriatic model mice [67]. Collectively, these findings indicate that VEGF is involved in aggravation of itch in psoriasis.

#### 4.4.2. Prostaglandin E2 (PGE_2_)

Prostaglandin E2 (PGE_2_) is a member of the prostanoids family and is synthesized from prostaglandin H2 (PGH_2_), a metabolite of arachidonic acid produced by a reaction catalyzed by cyclooxygenase-1 and -2 [142,143]. PGE_2_ concentrations were found to be elevated in tissue fluid from many inflammatory dermatoses, including psoriasis [69]. In addition to its vasodilatory effects, an intradermal injection of PGE_2_ induced weak itch in human subjects, and PGE_2_ also aggravated histamine- and serotonin-induced itch [70,71]. Since an intradermal injection of PGE_2_ did not induce itch-related scratching behavior in mice [144] and because the topical application of PGE_2_ inhibited spontaneous scratching in NC/Nga mice with chronic dermatitis [145], the underlying mechanisms in animals may not be applicable to humans. However, this lipid mediator may be involved in the induction and enhancement of itch in psoriasis in humans [102].

#### 4.4.3. Endothelin-1 (ET-1)

Endothelin-1 (ET-1) is a 21-amino acid peptide that is expressed by various cells, including endothelial cells, neurons, immune cells, and keratinocytes, and is a potent vasoconstrictor [146]. ET-1 exerts several biological functions in the skin, such as keratinocyte proliferation, leukocyte migration, and angiogenesis, which are also characteristic features of psoriasis [146]. This peptide has also been shown to shift the dendritic cell-T-cell response toward Th17/Th1 polarization [72]. The topical application of an ET-1 receptor (ET-A receptor) antagonist attenuated imiquimod-induced psoriasiform skin inflammation, including the phenotypic and functional activation of dendritic cells [146]. In patients with psoriasis, the expression of ET-1 was also more widely observed in lesional skin than in normal skin. Moreover, its expression level was slightly higher in severe cases than in mild or moderate cases [72]. A previous study showed that ET-1 induced a burning itch sensation when injected intradermally into humans [73]. In addition, ET-1 induced scratching behavior in a dose-dependent manner in mice and this scratching behavior was significantly reduced by an ET-A receptor antagonist, indicating that ET-1 mainly induces itch through ET-A receptors [147,148]. These findings imply that ET-1 acts as an itch inducer in the pathology of psoriasis.

#### 4.4.4. Cell Adhesion Molecules

In addition to the factors described above, several cell adhesion molecules are thought to be involved in itch-related events, such as the aggravation and modulation of psoriatic itch. An increase has been reported in the density of E-selectin–positive venules in patients with psoriasis, and the density of these venules correlated with the severity of pruritus [35]. Moreover, psoriatic patients with pruritus were found to have elevated serum levels of soluble vascular adhesion protein 1 (VAP-1) [74].

E-selectin, which is also known as CD62E or endothelial leukocyte adhesion molecule 1 (ELAM-1), and VAP-1 are adhesion molecules that promote leukocyte adhesion to the vasculature in order to induce inflammation [74,149]. Although the mechanism of itch in these molecules is currently unknown, angiogenesis induced by the Koebner phenomenon, which is the aggravation of inflammation by the itch-scratch cycle, has been suggested [35].

### 4.5. Epidermal Keratinocytes

A well-known factor contributing to the pathogenesis of psoriasis is abnormal keratinocyte proliferation [150]. Furthermore, the invasion of nerve endings occurs in the epidermal layer with psoriasis [35,89]. In these cases, the initial site of itch (i.e., nerve endings of peripheral sensory neurons) is likely to be adjacent to the layer of keratinocytes. Therefore, the contribution of keratinocytes to itch in psoriasis is considered to be significant. A previous study reported that keratinocytes in psoriatic patients with pruritus consistently showed the upregulated expression of each receptor for SP, CGRP, and NGF (NK-1R. CGRPR, and TrkA, respectively) [7]. We also demonstrated the expression of MOR, KOR, and their ligands in keratinocytes, with that of KOR and its ligand dynorphin-A being downregulated in psoriatic skin [41]. Among TRP channels, the gene expression of TRPV1, TRPM8, and TRPV3 was significantly upregulated in the epidermis of psoriatic patients, and the expression level of the TRPV1 gene correlated with the severity of psoriatic itch [58]. Importantly, NGF, which is produced by keratinocytes, acts as a neurotrophic factor in skin [151]. In addition, the expression of IL-31, TSLP, and α-MSH was shown to be upregulated in the psoriatic epidermis (keratinocytes) [49,58,62]. These findings indicate that epidermal keratinocytes sometimes promote itch directly, but mostly indirectly.

### 4.6. Others

This section summarizes itch modulators that affect the degree of pruritus in psoriasis.

#### 4.6.1. Dipeptidyl Peptidase IV (DPPIV, CD26)

CD26 is a 110 kDa multifunctional glycoprotein that is expressed on various cell types. This protein exhibits DPPIV enzymatic activity in its extracellular domain and is capable of cleaving the *N* terminus of peptides with l-proline or l-alanine at the penultimate position [152,153,154]. The enzyme is involved in the activation/inactivation of a number of cytokines, chemokines, and neuropeptides [155]. We and other groups recently demonstrated that DPPIV levels were elevated in the serum [36] and lesional skin [75] of psoriatic patients. In the imiquimod-induced murine model, we also reported that this enzyme cleaved SP to aggravate itch [75]. Notably, the level of the truncated form of SP cleaved by DPPIV was significantly elevated in the sera of patients with psoriasis [75]. In addition, the DPPIV inhibitor sitagliptin inhibited scratching behavior in psoriasis model mice [75]. Based on these findings, the DPPIV enzyme aggravates itch induced by SP in psoriasis.

#### 4.6.2. Lipocalin-2 (LCN2)

Lipocalin-2 (LCN2), also known as neutrophil gelatinase-binding protein (NGAL), is a 25 kDa protein that is mainly secreted by activated neutrophils [156]. This protein is a critical iron regulatory protein under physiological and inflammatory conditions [157]. A previous study using an atopic dermatitis murine model (NC/Nga mice) revealed that LCN2 produced by activated astrocytes in the spinal cord enhanced itch induced by GRP, an itch-specific neuropeptide [77]. We recently demonstrated that serum levels of LCN2 were elevated in patients with psoriasis, and that this increase correlated with the severity of pruritus [76]. Based on these findings, LCN2 may also be partially involved in the aggravation of pruritus in psoriasis.

## 5. Ongoing and Future Trials

Information on clinical trials or studies that are currently underway is described herein (summarized in Table 2). Among itch mediators in the nervous system (Section 4.1), a phase II randomized clinical trial has been conducted on the NK-1R (a receptor for SP) inhibitor, serlopitant [158]. When 204 psoriatic patients were randomized to receive 5 mg serlopitant or a placebo orally once daily for 8 weeks, response rates at 4 and 8 weeks were significantly higher in the serlopitant-treated group than in the placebo-treated group [158]. These findings suggest that neurogenic inflammation caused by SP may be an important factor contributing to psoriatic itch and that serlopitant is an effective therapeutic option.

Another randomized clinical trial (phase IIb) on itch mediators in the nervous system was conducted with CT327, a topical TrkA (a receptor for NGF) kinase inhibitor. Although no effects were observed on disease severity, clinically significant reductions in pruritus were observed in psoriatic patients with moderate pruritus, which was treated with CT237 ointment at the lowest dose (0.05%) [98]. Another phase IIb trial was conducted on another TrkA inhibitor, SNA-120; however, no significant effects were observed because SNA-120 and the vehicle both exerted strong anti-pruritic effects [159].

A phosphodiesterase-4 (PDE4) inhibitor is also an antipruritic drug that may affect the nervous system. Apremilast, a DPE4 inhibitor, is currently at phase IV in a clinical trial for pruritus in patients with scalp psoriasis and at phase III in that for plaque psoriasis [159,160]. PDE4 is an enzyme that digests cAMP into inactive AMP, and its inhibitor has been shown to alleviate psoriatic symptoms mainly by its anti-inflammatory effects [161]. However, PDE4 inhibitors do not induce the degranulation of mast cells, the main source of inflammation-mediated itch [165], and the PDE4 inhibitor E6005 has been reported to exhibit acute antipruritic activity [166]. These findings suggest that PDE4 inhibitors exert acute antipruritic effects without mediating anti-inflammatory effects. Moreover, E6005 was shown to exert inhibitory effects on the TRPV1-mediated depolarization activity of C-fibers, potentially by increasing cAMP levels, suggesting that this drug exerts direct effects on nerves [167]. Although a phase IV clinical trial on scalp psoriasis is ongoing, the marked attenuation of pruritus, as measured by VAS, has been reported in patients with plaque psoriasis receiving apremilast [159].

Clinical trials on antipruritic drugs targeting itch mediators and exacerbating factors, which are associated with the immune system (see Section 4.2), are also underway. Although the findings obtained have not yet been reported, the efficacy of KPL-716, a monoclonal antibody against OSMRβ (oncostatin M receptor β, a subunit for IL-31 receptor), was evaluated in a pilot phase II study on patients with disease-accompanying itch, including those with plaque psoriasis [159]. In addition, promising findings from clinical trials have been obtained for two IL-17A antibodies. Secukinumab, a selective monoclonal antibody, and ixekizumab, another monoclonal antibody that also targets IL-17A, are currently being examined in clinical trials. In two phase III clinical trials, the attenuation of psoriatic itch was found to be significantly greater with secukinumab than with a placebo or the TNF-α inhibitor, etanercept [110,162]. Furthermore, in clinical trials, better outcomes were obtained with secukinumab than with the anti-Il-12/IL 23p40 monoclonal antibody, ustekinumab [116]. In a phase III trial, the attenuation of itch was greater with ixekizumab than with a placebo or etanercept as early as the first week [163], and excellent outcomes were reported in a long-term extension study [108]. Although its underlying mechanism currently remains unclear, the findings of these clinical trials suggest that IL-17 antibodies attenuate psoriatic itch. Furthermore, in two randomized phase III trials, the oral JAK inhibitor tofacitinib was shown to attenuate itch in psoriatic patients shortly after the start of treatment [164]. As an underlying mechanism for this effect, Hashimoto et al. showed that tofacitinib significantly reduced the mRNA expression levels of IL-22, IL-23, and IL-31 and also increased the density of peptidergic epidermal nerve fibers [168].

However, clinical trials have not yet been conducted on itch mediators of psoriatic itch other than those listed above. As further research is conducted on itch in psoriasis, more drug candidates, including those used to treat other diseases, may be subjected to clinical trials and become available for the treatment of psoriatic itch.

## 6. Conclusions

In this review, we discussed a number of clinical factors and molecular and cellular mechanisms that are considered to be involved in induction or sensitization of psoriatic itch. As described earlier, accumulating evidence shows that psoriatic patients consider pruritus to be not only the most commonly subjective sensation, but also one of the most bothersome symptoms of this disease. Furthermore, the severity of pruritus has been often correlated with that of psoriasis. However, even if other symptoms diminish, itch often permanently persists. This difficulty of management may be due to a wide variety of itch mediators and/or modulators being involved in the pathogenesis of psoriatic itch as a result of complex interactions between the nervous, immune, neuroendocrine, and vascular systems in the pathology of psoriasis. Unfortunately, due to the delay in recognizing the severity of pruritus in psoriasis, the development of therapeutic options has been delayed. However, the medications for psoriatic itch will be more developed in the future, as in the case with biologics and JAK inhibitors in atopic dermatitis.

## Figures and Tables

**Table 1:** Potential mediators of itch in psoriasis.

System	Category	Mediator	Expression Changes in the Mediator	Mechanisms (Such as Receptors)	Expression Changes in Receptors of the Mediator	Predictive Effects on Itch
Nervous	Neuropeptides	SP	↑ (L [35], B [36])	NK-1R	↑ (L [7])	Induction of itch [37,38]
				MrgprX2(Hu)	-	
				MrgprB2(Ms)	-	
				MrgprA1(Ms)	-	
		CGRP	↑ (B [34])	CGRPR	↑ (B [7])	Aggravation of itch? [39]
		NPY	↓ (B [40])	NPY1R	-	Suppression of mechanical itch [25]
	Opioids	β-endorphin	UC [41]/↑ (L [42])	MOR	UC [41]	Induction of itch [43]
		Dynorphin A	↓ (L [41])	KOR	↓ (L [41])	Suppression of itch [42,43]
	Neurotrophins	NGF	↑ (L [35,44])	TrkA	↑ (L [35])	NGF-TrkA axis: aggravation of histamine-independent itch [45]
				P75	-	
Immune	Cytokines	IL-31	↑ (L [46], B [47])	IL-31RA	-	Induction of itch [48]
				OSMRβ	-	
		TSLP	↑ (L [49], B [50])	TSLPR,	-	Induction of itch [51]
				IL-7Rα	-	
		IL-2	↑ (L [35])	IL-2Rα	-	Induction of itch [52,53,54]
				IL-2Rβ	-	
				IL-2Rγ	-	
		IL-17	↑ (L [21])	IL-17Rs	-	Enhancement of itch by altering perception? [55]
		IL-22	↑ (L [56])	IL-22R1	-	Enhancement of itch by activation of the GRP-GRPR signal? [57]
				IL-10R2	-	
		IL-23	↑ (L [56,58])	IL-23R	-	Enhancement of itch though the aggravation of inflammation? [59]
				IL-12Rβ1	-	
		IL-26	↑ (L [60])	Il-20R1	-	Enhancement of itch by promoting the sensory neuronal development? [61]
				IL-10R2	-	
Endocrine	HPA axis	CRH	↑ (L [62])	CRHR1	-	Induction/aggravation of itch by mast cell degranulation [63,64]
		α-MSH	↑ (L [62])	MC1R	-	Induction of itch [65,66]
				MC5R		
Vascular	Growth factors	VEGF	↑ (L [67], B [68])	VEGFRs	-	Aggravation of itch? [67]
	Prostanoids	PGE2	↑ (L [69])	cAMP	-	Induction of weak itch and enhancement of histamine-/serotonin- induced itch [70,71]
	Autacoids	ET-1	↑ (L [72])	ET-A/ET-B	-	Induction of itch [73]
	Cell adhesion molecules	E-selectin	↑ (L [35])	-	-	Aggravation of itch?
		VAP-1	↑ (B [74])	-	-	Aggravation of itch?
Others	Peptidases	DPPIV	↑ (L [36], B [75])	SP (cleavage)	↑ (L [35], B [36])	Aggravation of itch [39,75]
	Lipocalins	LCN2	↑ (B [76])	GRP (production)	-	Aggravation of itch [77]

↑; upregulation, ↓; downregulation, L; lesional skin, B; blood (serum or plasma), UC; unchanged, Hu; human Ms; mouse, -; unknown.

**Table 2:** Candidates for psoriatic antipruritics in clinical trials.

Category	Drug Name	Target Interaction	Phase	Administration Type	Significant Findings	NCT#	References
NK-1R inhibitor	Serlopitant	NK-1R	2	Oral	Yes	NCT03343639	[158]
TrkA inhibitor	CT327	TrkA	2b	Topical	Yes	NCT01465282	[98]
	SNA-120		2b	Topical	No *	NCT03322137	[159]
PDE4 inhibitor	Apremilast	PDE4	Scalp: 4	Oral	-	NCT03553433	[159,160]
			Plaque: 3	Oral	Yes	NCT03721172	
OSMRβ moAb	KPL-716	OSMRβ	Plaque: 2 (pilot study)	Injection	-	NCT03858634	[161]
IL-17 moAb	Secukinumab	IL-17A	3	SC	Yes	NCT01365455	[110]
			3	SC	Yes	NCT01358578	[162]
	Ixekizumab	IL-17A	3	SC	Yes	NCT01597245	[163]
			3 (long-term test)	SC	Yes	NCT01474512	[108]
JAK inhibitor	Tofacitinib	JAK-STAT pathway	3	Oral	Yes	NCT01276639	[164]
					Yes	NCT01309737	

* Phase 3 trials have been decided. NCT#; national clinical trial number, Scalp; scalp psoriasis, Plaque; plaque psoriasis, ―; unknown, moAb; monoclonal antibody, SC; subcutaneous injection.

## Abbreviations

α-MSH	α-melanocyte-stimulating hormone
CGRP	Calcitonin gene-related peptide
CRH	Corticotropin-releasing hormone
DPPIV	Dipeptidyl peptidase IV
DRG	Dorsal root ganglion
ET-1	Endothelin-1
GRP	Gastrin-releasing peptide
GABA	Gamma-amino butyric acid
HPA	Hypothalamic-pituitary-adrenal
IL	Interleukin
JAK	Janus kinase
LCN2	Lipocalin-2
KOR	κ-opioid receptor
MC1R	Melanocortin 1 receptor
MC5R	Melanocortin 5 receptor
MOR	μ-opioid receptor
Mrgpr	Mas-related G protein-coupled receptor
NGF	Nerve growth factor
NK-1R	Neurokinin-1 receptor
NPY	Neuropeptide Y
OSMR	Oncostatin M receptor
p75	p75 neurotrophin receptor
PGA	Physician’s Global Assessment
PGE_2_	Prostaglandin E2
SP	Substance P
STAT	Signal transducer and activator of transcription
TrkA	Tropomyosin-receptor kinase A
TRP	Transient receptor potential
TRPA	Transient receptor potential ankyrin
TRPC	Transient receptor potential canonical
TRPM	Transient receptor potential melastatin
TRPV	Transient receptor potential vanilloid
TSLP	Thymic stromal lymphoprotein
Th	T helper type
VAP-1	Vascular adhesion protein 1
VAS	Visual Analog Scale
VEGF	Vascular endothelial growth factor
